# Supplementary material for: Pharmacological Treatments for Cocaine Craving: What Is the Way Forward? A Systematic Review
Source: Brain Sci. 2022 Nov 14;12(11):1546. doi: 10.3390/brainsci12111546 (PMC9688748; doi:10.3390/brainsci12111546)
Supplement: Supplementary file 1 [file brainsci-12-01546-s001.zip › brainsci-1850935-supplementary.pdf]

SUPPLEMENTARY MATERIAL

Search strategy in Scholar Google

The saturation strategy used in Google Scholar search was two consecutive pages without studies in the scope of the review; of the total of 18.200 results encountered, distributed by 10 per page and ordered by relevance.

Table S1. - Structured cocaine craving scales.

SCALE	ITEMS	DIMENSIONS	TIME EVALUATION
Cocaine Craving Questionnaire Brief (CCQ-B)	10 items	1) Desire to use cocaine;<br>2) Intentions and planning to use cocaine;<br>3) Anticipation of positive outcome;<br>4) Anticipation of relief of withdrawal or dysphoria;<br>5) Lack of control over use.	At the present moment
Cocaine Craving Questionnaire-Now (CCQ-Now)	45 items	1) Desire to use cocaine;<br>2) Intentions and planning to use cocaine;<br>3) Anticipation of positive outcome;<br>4) Anticipation of relief of withdrawal or dysphoria;<br>5) Lack of control over use.	At the present moment
Brief Substance Craving Scale (BSCS)	Multidrugs; 4 items	1) Intensity;<br>2) Frequency;<br>3) Length.	Past 24 hours
(Minnesota) Cocaine Craving Scale (CCS/MCCS)	5 items	1) Intensity;<br>2) Frequency;<br>3) Length;<br>4) Evolution of craving;<br>5) Evolution of treatment.	Past Week
Cocaine Selective Severity Assessment (CSSA)	18 items of symptoms (2 for craving)	1) Craving frequence;<br>2) Craving intensity.	Past 24 hours
Voris Cocaine Craving Questionnaire (VCCQ)	4 items	1) Craving intensity;<br>2) Mood;<br>3) Energy;<br>4) Sick feelings	Smelson DA, McGee-Caulfield E, Bergstein P, et al. Initial validation of the Voris Cocaine Craving Scale: a preliminary report. J Clin Psychol. 1999;55(1):135–139.
Questionnaires of cocaine craving and urges (QCU)	33 items	1) Desire to use;<br>2) Intention to use;<br>3) Antecipation of positive outcome;<br>4) Antecipation of reliefe of negative emotional states	At the present moment
Cocaine Urge Questionnaire (CUQ)	8 items	NA	At the present moment
Quantitative Cocaine Inventory (QCI)	5 items	1) Frequency, amount and route of cocaine use;<br>2) Cocaine craving, quality of high, and amount of control over craving.	During past week
Desire for Drug Questionnaire (DDQ)	NA	1) Desire and intention;<br>2) Negative reinforcement;<br>3) Control	NA

Obsessive Compulsive Drug Use Scale (OCDUS)	12 items	1) Thoughts about drug and interference;<br>2) Desire and control;<br>3) Resistance to thoughts and intention.	NA

NA: Not available

Table S2. Results of the quality/bias assessment using the Cochrane Collaboration tool for assessing risk of bias.

Author, Year, Country	Random sequence generation	Allocation concealment	Blinding of participants and personnel	Blinding of outcome assessment	Incomplete outcome data	Selective reporting	Score: based on the number of domains classified as low risk
Afshar et. al., 2012, USA [19]	Unclear bias risk	High bias Risk	Unclear bias risk	High bias risk	Low bias risk	Low bias risk	6
Akerele et. al., 2007, USA [65]	Low bias risk	High bias Risk	Unclear bias risk	High bias risk	Unclear bias risk	Low bias risk	6
Alim et. al., 1995, USA[37]	Unclear bias risk	Unclear bias risk	Unclear bias risk	High bias risk	Low bias risk	Low bias risk	7
Anderson et. al., 2009, USA [38]	Low bias risk	Low bias risk	Unclear bias risk	High bias risk	Low bias risk	Low bias risk	9
Arndt et. al., 1992, USA [20]	Low bias risk	High bias risk	Low bias risk	High bias risk	Low bias risk	Low bias risk	8
Batki et. al., 1996, USA [21]	Unclear bias risk	High bias Risk	Unclear bias risk	High bias risk	Low bias risk	Low bias risk	6
Becker et. al., 2020, USA [88]	Low bias risk	Low bias risk	Low bias risk	High bias risk	Low bias risk	Low bias risk	10
Beresford et al., 2017, USA [66]	Low bias risk	High bias Risk	Low bias risk	High bias risk	Low bias risk	Low bias risk	8
Bisaga et. al., 2006, USA [77]	Low bias risk	Low bias risk	Unclear bias risk	High bias risk	Unclear bias risk	High bias risk	6
Bisaga et. al., 2010, USA [89]	Unclear bias risk	Unclear bias risk	Unclear bias risk	High bias risk	Low bias risk	Low bias risk	7
Brown et. al., 2012, USA [78]	Low bias risk	Low bias risk	Unclear bias risk	High bias risk	Low bias risk	Low bias risk	9
Brown et. al., 2015, USA [90]	Low bias risk	Low bias risk	Low bias risk	High bias risk	Low bias risk	Low bias risk	10
Buydens-Branchey et. al., 1997, USA [39]	Unclear bias risk	High bias Risk	Unclear bias risk	High bias risk	Low bias risk	Low bias risk	6
Buydens-Branchey et. al., 1998, USA [22]	High bias risk	High bias Risk	Unclear bias risk	High bias risk	Low bias risk	Low bias risk	5
Campbell et. al., 2003, USA [23]	Unclear bias risk	Unclear bias risk	Low bias risk	High bias risk	Unclear bias risk	Unclear bias risk	6
Cantilena et. al., 2012, USA [40]	Low bias risk	Low bias risk	Low bias risk	High bias risk	Low bias risk	Low bias risk	10
Chadwick et. al., 1990, USA [91]	Unclear bias risk	High bias Risk	Unclear bias risk	High bias risk	Low bias risk	Low bias risk	6
Ciraulo, Sarid-Segal et. Al., 2005, USA [25]	Unclear bias risk	High bias Risk	Unclear bias risk	High bias risk	Low bias risk	Low bias risk	6
Ciraulo, Knapp et. al., 2005, USA [24]	Unclear bias risk	Unclear bias risk	Low bias risk	High bias risk	Low bias risk	Low bias risk	8
Cornish et. al., 2001, USA [41]	Unclear bias risk	High bias Risk	Unclear bias risk	High bias risk	Low bias risk	Low bias risk	6

Crosby et. al., 1996, USA [79]	Unclear bias risk	High bias Risk	Low bias risk	Low bias risk	Low bias risk	Low bias risk	9
Dackis et. al., 1987, USA [92]	Unclear bias risk	High bias Risk	Unclear bias risk	High bias risk	Unclear bias risk	Unclear bias risk	4
Dackis et. al., 2003, USA [42]	Unclear bias risk	High bias Risk	Unclear bias risk	High bias risk	Unclear bias risk	Unclear bias risk	4
Dackis et. al., 2005, USA [43]	Low bias risk	Low bias risk	Unclear bias risk	High bias risk	Low bias risk	Low bias risk	9
Dackis et. al., 2012, USA [44]	Low bias risk	Low bias risk	Unclear bias risk	High bias risk	Low bias risk	Low bias risk	9
Dakwar, Levin et al., 2014, USA [93]	Low bias risk	Unclear bias risk	Low bias risk	Unclear bias risk	Low bias risk	Low bias risk	9
Dakwar, Anerella et. al., 2014, USA [94]	Unclear bias risk	High bias Risk	Unclear bias risk	High bias risk	Unclear bias risk	Low bias risk	5
Dakwar et. al., 2016, USA [95]	Low bias risk	Unclear bias risk	Low bias risk	High bias risk	Low bias risk	Low bias risk	9
Dieckmann et. al., 2014, Brazil [96]	Low bias risk	Low bias risk	Unclear bias risk	Unclear bias risk	Low bias risk	Low bias risk	10
Ehrman et. al., 1996, USA [45]	Unclear bias risk	Unclear bias risk	Unclear bias risk	High bias risk	Unclear bias risk	Unclear bias risk	5
Eiler et. al., 1995, USA [97]	Unclear bias risk	High bias Risk	Low bias risk	High bias risk	Low bias risk	Low bias risk	7
Elkashef et. al., 2006, USA [98]	Low bias risk	Low bias risk	Unclear bias risk	High bias risk	Low bias risk	Low bias risk	9
Fox et. al., 2012, USA [99]	Unclear bias risk	High bias Risk	Unclear bias risk	High bias risk	Low bias risk	Low bias risk	6
Fox et. al., 2013, USA [100]	Low bias risk	Low bias risk	Low bias risk	High bias risk	Low bias risk	Low bias risk	10
Gawin et. al., 1989, USA [26]	Unclear bias risk	High bias Risk	Unclear bias risk	High bias risk	Unclear bias risk	Low bias risk	5
Goudriaan et. al, 2013, Netherlands [46]	Unclear bias risk	High bias Risk	Unclear bias risk	High bias risk	Low bias risk	Low bias risk	6
Grabowski et. al., 1995, USA [27]	Study 1: Unclear bias risk<br>Study 2: Unclear bias risk	Study 1: Unclear bias risk<br>Study 2: Unclear bias risk	Study 1: Low bias risk<br>Study 2: Low bias risk	Study 1: High bias risk<br>Study 2: High bias risk	Study 1: Low bias risk<br>Study 2: Unclear bias risk	Study 1: Low bias risk<br>Study 2: Unclear bias risk	Study 1: 8<br>Study 2: 6
Grabowski et. al., 1997, USA [47]	Unclear bias risk	High bias Risk	Unclear bias risk	High bias risk	Unclear bias risk	High bias risk	3
Greenwald et. al., 2010, USA [48]	Unclear bias risk	Unclear bias risk	Unclear bias risk	High bias risk	Low bias risk	Low bias risk	7
Halikas et. al., 1997, USA [80]	Unclear bias risk	Low bias risk	Low bias risk	Unclear bias risk	Low bias risk	Low bias risk	10
Hamilton et. al., 2009, USA [67]	Low bias risk	High bias Risk	Unclear bias risk	High bias risk	High bias risk	Unclear bias risk	4
Handelsman et. al., 1995, USA [101]	Unclear bias risk	Unclear bias risk	Unclear bias risk	High bias risk	Low bias risk	Low bias risk	7
Handelsman et. al., 1997, USA [102]	Unclear bias risk	High bias Risk	Unclear bias risk	High bias risk	Low bias risk	Low bias risk	6
Harris et. al., 2004, USA [28]	Unclear bias risk	Unclear bias risk	Unclear bias risk	High bias risk	Low bias risk	Low bias risk	7
Jobes et. al., 2011, USA [103]	Unclear bias risk	Low bias risk	Unclear bias risk	High bias risk	Low bias risk	Low bias risk	8
Jobes et. al.; 2015; USA [104]	Low bias risk	Low bias risk	Unclear bias risk	High bias risk	Unclear bias risk	Unclear bias risk	7
Johnson et. al., 1997, USA [50]	Unclear bias risk	High bias Risk	Low bias risk	High bias risk	Low bias risk	Low bias risk	6
Johnson et. al., 2004, USA [105]	Unclear bias risk	High bias Risk	Unclear bias risk	High bias risk	Low bias risk	Low bias risk	6

Johnson et. al., 2012, USA [81]	Unclear bias risk	High bias Risk	Low bias risk	High bias risk	Low bias risk	Low bias risk	7
Johnson et. Al., 2019, USA [106]	Low bias risk	Low bias risk	Unclear bias risk	High bias risk	Unclear bias risk	Unclear bias risk	7
Johnson et. al., USA, 2013 [87]	Low bias risk	Low bias risk	Low bias risk	High bias risk	Unclear bias risk	Low bias risk	9
Kablinger et. al., 2012, USA [107]	Low bias risk	High bias Risk	Unclear bias risk	High bias risk	Low bias risk	Low bias risk	7
Kampman et. al., 2015, USA [51]	Low bias risk	High bias risk	Unclear bias risk	High bias risk	Low bias risk	Low bias risk	7
Kampman et. al., 2001, USA [108]	Low bias risk	High bias risk	Unclear bias risk	High bias risk	Unclear bias risk	Unclear bias risk	5
Kampman et. al., 2003, USA [68]	Low bias risk	High bias risk	Unclear bias risk	High bias risk	Low bias risk	Low bias risk	7
Kampman et. al., 2006, USA [109]	Unclear bias risk	High bias Risk	Low bias risk	Low bias risk	Unclear bias risk	Unclear bias risk	7
Kampman et. al., 2010, USA [147]	Low bias risk	Low bias risk	Unclear bias risk	High bias risk	Low bias risk	Low bias risk	10
Kampman et. al., 2013, USA [83]	Unclear bias risk	Unclear bias risk	Unclear bias risk	High bias risk	Unclear bias risk	High bias risk	4
Karila et. Al, 2016, France [52]	Unclear bias risk	High bias Risk	Unclear bias risk	High bias risk	Low bias risk	Low bias risk	6
Kolar et. al., 1992, USA [29]	Unclear bias risk	Unclear bias risk	Low bias risk	High bias risk	Low bias risk	Low bias risk	8
Kosten et. al., 1992, USA [30]	Unclear bias risk	High bias Risk	Low bias risk	High bias risk	Unclear bias risk	Unclear bias risk	5
Larowe et. al., 2013, USA [148]	Low bias risk	Low bias risk	Unclear bias risk	Unclear bias risk	Low bias risk	Low bias risk	10
Lee at. Al., 2015, USA [111]	Unclear bias risk	Unclear bias risk	Unclear bias risk	High bias risk	Low bias risk	Low bias risk	7
Levin et. al., 2007, USA [53]	Unclear bias risk	High bias Risk	Unclear bias risk	High bias risk	Unclear bias risk	Unclear bias risk	4
Leyton et. al., 2005, USA [112]	Unclear bias risk	High bias Risk	Unclear bias risk	High bias risk	Low bias risk	Low bias risk	6
Licata et. al., 2011, USA [113]	Unclear bias risk	High bias Risk	Unclear bias risk	High bias risk	Low bias risk	Low bias risk	6
Loebl et. al., 2008, USA [69]	Unclear bias risk	High bias Risk	Unclear bias risk	High bias risk	Low bias risk	Low bias risk	6
Lofwall et. al., 2014, USA [70]	Unclear bias risk	Low bias risk	Low bias risk	High bias risk	Unclear bias risk	High bias risk	6
Malcolm et. al., 2000, USA [114]	Unclear bias risk	Unclear bias risk	Unclear bias risk	High bias risk	Unclear bias risk	Unclear bias risk	5
Malcolm et. al., 2005, USA [115]	Unclear bias risk	High bias Risk	Unclear bias risk	High bias risk	Low bias risk	Low bias risk	6
McDowell et. al, 2005, USA [31]	Low bias risk	Low bias risk	Low bias risk	High bias risk	Low bias risk	Low bias risk	10
Milivojevic et. Al., 2016, USA [117]	Low bias risk	Low bias risk	Unclear bias risk	High bias risk	Low bias risk	Low bias risk	9
Modesto-Lowe, 1997, USA [118]	Unclear bias risk	High bias Risk	Unclear bias risk	High bias risk	Low bias risk	Low bias risk	6
Mooney et. al., 2006, USA [120]	Unclear bias risk	Unclear bias risk	Unclear bias risk	High bias risk	High bias risk	High bias risk	3
Mooney et. al., 2009, USA [54]	Unclear bias risk	High bias Risk	Unclear bias risk	High bias risk	Low bias risk	Low bias risk	6
Mooney et. al., 2015, USA [55]	Low bias risk	Low bias risk	Unclear bias risk	High bias risk	Low bias risk	Low bias risk	9
Moran et al., 2017, USA [71]	Low bias risk	Unclear bias risk	Low bias risk	High bias risk	Unclear bias risk	Low bias risk	8
Moran-Santa Maria et. al., 2015, USA [121]	Low bias risk	High bias Risk	Low bias risk	Unclear bias risk	Low bias risk	Low bias risk	9

Nann-Vernotica et. al., 2001, USA [122]	Unclear bias risk	High bias risk	Unclear bias risk	High bias risk	Unclear bias risk	Unclear bias risk	4
Nasser et. al., 2014, USA [123]	Low bias risk	Low bias risk	Unclear bias risk	High bias risk	Unclear bias risk	Unclear bias risk	7
Newton at. Al., 2015, USA [124]	Unclear bias risk	High bias Risk	Unclear bias risk	High bias risk	Low bias risk	Low bias risk	6
Nuijten et. al., 2016, Netherlands [56]	Low bias risk	Low bias risk	Unclear bias risk	Low bias risk	Low bias risk	Low bias risk	11
Oliveto et. al., 1995, USA [33]	Unclear bias risk	High bias Risk	Low bias risk	High bias risk	Low bias risk	Low bias risk	7
Passos et. al., 2005, Brazil [34]	Low bias risk	Unclear bias risk	Unclear bias risk	High bias risk	Unclear bias risk	Unclear bias risk	6
Perry et. al., 2004, USA [57]	Unclear bias risk	High bias Risk	Unclear bias risk	High bias risk	Low bias risk	Low bias risk	6
Petrakis et. al., 2000, USA [125]	Unclear bias risk	High bias Risk	Unclear bias risk	High bias risk	Unclear bias risk	Unclear bias risk	4
Pirtle et al., 2019, USA [58]	Low bias risk	Low bias risk	Unclear bias risk	High bias risk	Low bias risk	Low bias risk	9
Plebani et. al., 2012, USA [126]	Unclear bias risk	High bias Risk	Unclear bias risk	High bias risk	Low bias risk	Low bias risk	6
Preston et. al., 1993, USA [127]	Unclear bias risk	High bias Risk	Unclear bias risk	High bias risk	Low bias risk	Low bias risk	7
Price et. al., 2012, USA [128]	Unclear bias risk	Low bias risk	Low bias risk	High bias risk	Low bias risk	Low bias risk	9
Prisciandaro et. al., 2013, USA [129]	Unclear bias risk	High bias Risk	Unclear bias risk	Unclear bias risk	Unclear bias risk	Unclear bias risk	5
Reid et. al., 1998, USA [145]	Low bias risk	High bias Risk	Unclear bias risk	High bias risk	Low bias risk	Low bias risk	7
Reid et. al., 1999, USA [85]	Low bias risk	Low bias risk	Unclear bias risk	High bias risk	Low bias risk	Low bias risk	9
Reid, Angrist et. al., 2005, USA [130]	Low bias risk	High bias Risk	Low bias risk	High bias risk	Low bias risk	Low bias risk	8
Reid, Casadonte et. al., 2005, USA [72]	Low bias risk	High bias Risk	Low bias risk	High bias risk	Low bias risk	Low bias risk	8
Reid et. al., 2006, USA [131]	Unclear bias risk	Unclear bias risk	Low bias risk	High bias risk	Low bias risk	Low bias risk	8
Reid et. al., 2009, USA [84]	Low bias risk	Low bias risk	Unclear bias risk	High bias risk	Low bias risk	Low bias risk	9
Renshaw et. al., 1999, USA [132]	Unclear bias risk	High bias Risk	Unclear bias risk	High bias risk	Low bias risk	Low bias risk	6
Roache et. al., 2000, USA [59]	S1: Unclear risk bias<br>S2: High bias risk	S1: Unclear risk bias<br>S2: Unclear risk bias	S1: Unclear risk bias<br>S2: Unclear risk bias	S1: High risk bias<br>S2: High risk bias	S1: Low bias risk<br>S2: Unclear bias risk	S1: Low bias risk<br>S2: Unclear bias risk	S1:7<br>S2: 4
Rosse et. al., 1994, USA [133]	Unclear bias risk	High bias Risk	Unclear bias risk	High bias risk	Low bias risk	Low bias risk	6
Saladin et. al., 2013, USA [134]	Low bias risk	Low bias risk	Low bias risk	Low bias risk	Low bias risk	Low bias risk	12
Santa Ana et. al., 2015, USA [135]	Unclear bias risk	High bias Risk	Unclear bias risk	High bias risk	Low bias risk	Low bias risk	6
Sayers et. al., 2005, USA [73]	Unclear bias risk	High bias Risk	Unclear bias risk	High bias risk	Low bias risk	Low bias risk	6
Schmitz et al., 2017 , USA [136]	Low bias risk	High bias Risk	Unclear bias risk	Low bias risk	Unclear bias risk	Unclear bias risk	7
Schmitz et. al., 2001, USA [137]	Unclear bias risk	Unclear bias risk	Unclear bias risk	High bias risk	Low bias risk	Low bias risk	7
Schmitz et. al., 2008, USA [138]	Low bias risk	Low bias risk	Low bias risk	High bias risk	Low bias risk	Low bias risk	10
Schubiner et. al., 2002, USA [61]	Unclear bias risk	Low bias risk	Unclear bias risk	High bias risk	Unclear bias risk	Unclear bias risk	6

Schulte et al., 2018, Netherlands [139]	Low bias risk	Unclear bias risk	Low bias risk	Unclear bias risk	Low bias risk	Low bias risk	10
Shearer et. al., 2003, Australia [62]	Low bias risk	High bias Risk	Unclear bias risk	Low bias risk	Unclear bias risk	Low bias risk	8
Shoptaw et. al., 2002, USA [140]	Unclear bias risk	High bias Risk	Unclear bias risk	High bias risk	Low bias risk	Low bias risk	6
Shoptaw et. al., 2003, USA [141]	Unclear bias risk	High bias Risk	Unclear bias risk	High bias risk	High bias risk	High bias risk	2
Shoptaw et. Al., 2008, USA [35]	Unclear bias risk	High bias Risk	Unclear bias risk	High bias risk	Low bias risk	Low bias risk	6
Smelson et. al., 2004, USA [74]	Unclear bias risk	High bias Risk	Unclear bias risk	High bias risk	Low bias risk	Low bias risk	6
Smelson et. al., 2006, USA [75]	Unclear bias risk	Unclear bias risk	Unclear bias risk	High bias risk	Low bias risk	Low bias risk	7
Somoza et. al., 2013, USA [144]	Low bias risk	Low bias risk	Unclear bias risk	High bias risk	Unclear bias risk	Unclear bias risk	7
Stine et. al., 1995, USA [63]	Unclear bias risk	High bias Risk	Unclear bias risk	High bias risk	Low bias risk	Low bias risk	6
Tapp et. al., 2015, USA [76]	Low bias risk	Low bias risk	Unclear bias risk	High bias risk	Low bias risk	Low bias risk	9
Umbricht et. al., 2014, USA [86]	Low bias risk	Low bias risk	Low bias risk	High bias risk	Unclear bias risk	Unclear bias risk	8
Walsh et. al., 2013, USA [64]	Low bias risk	Low bias risk	Low bias risk	High bias risk	Unclear bias risk	Low bias risk	9
Winhusen, Somoza, Sarid-Segal et. al., 2007, USA [142]	Low bias risk	Low bias risk	Unclear bias risk	High bias risk	Low bias risk	Low bias risk	9
Winhusen et. al., 2005, USA [110]	Unclear bias risk	High bias Risk	Unclear bias risk	High bias risk	Unclear bias risk	Low bias risk	5
Winhusen, Somoza, Ciraulo et. al., 2007, USA [87]	Low bias risk	Low bias risk	Unclear bias risk	High bias risk	Low bias risk	Low bias risk	9
Winstanley et. al., 2005, USA [36]	Low bias risk	Low bias risk	Low bias risk	High bias risk	Unclear bias risk	Unclear bias risk	8
Yonkers et. al., 2014, USA [146]	Low bias risk	Low bias risk	Low bias risk	Low bias risk	Unclear bias risk	Unclear bias risk	10

19. Afshar, M.; Knapp, C.; Sarid-Segal, O.; Devine, E.; Colaneri, L.S.; Tozier, L.; Waters, M.E.; Putnam, M.A.; Ciraulo, D. The Efficacy of Mirtazapine in the Treatment of Cocaine Dependence with Comorbid Depression. Am. J. Drug Alcohol Abus. 2012, 38, 181–186. https://doi.org/10.3109/00952990.2011.644002.
20. Arndt, I.O.; Dorozynsky, L.; Woody, G.E.; McLellan, A.T.; O'Brien, C.P. Desipramine Treatment of Cocaine Dependence in Methadone-Maintained Patients. Arch. Gen. Psychiatry 1992, 49, 888–893. https://doi.org/10.1001/archpsyc.1992.01820110052008
21. Batki, S.L.; Washburn, A.M.; Delucchi, K.; Jones, R.T. A controlled trial of fluoxetine in crack cocaine dependence. *Drug Alcohol Depend.* **1996**, *41*, 137–142. https://doi.org/10.1016/0376-871601233-1.
22. Buydens-Branchey, L.; Branchey, M.; Hudson, J.; Rothman, M.; Fergeson, P.; McKernin, C. Effect of fenfluramine chal-lenge on cocaine craving in addicted male users. *Am. J. Addict.* **1998**, *7*, 142–155.
23. Campbell, J.; Nickel, E.J.; Penick, E.C.; Wallace, D.; Gabrielli, W.F.; Rowe, C.; Liskow, B.; Powell, B.J.; Thomas, H.M. Comparison of desipramine or carbamazepine to placebo for crack cocaine-dependent patients. *Am. J. Addict.* **2003**, *12*, 122–136.
24. Ciraulo, D.A.; Knapp, C.; Rotrosen, J.; Sarid-Segal, O.; Ciraulo, A.M.; LoCastro, J.; Greenblatt, D.J.; Leiderman, D. Nefazodone treatment of cocaine dependence with comorbid depressive symptoms. *Addiction* **2005**, *100*, 23–31. https://doi.org/10.1111/j.1360-0443.2005.00984.x.
25. Ciraulo, D.A.; Sarid-Segal, O.; Knapp, C.M.; Ciraulo, A.M.; LoCastro, J.; Bloch, D.A.; Montgomery, M.A.; Leiderman, D.B.; Elkashef, A. Efficacy screening trials of paroxetine, pentoxifylline, riluzole, pramipexole and venlafaxine in cocaine dependence. *Addiction* **2005**, *100*, 12–22. https://doi.org/10.1111/j.1360-0443.2005.00985.x.
26. Gawin, F.H.; Kleber, H.D.; Byck, R.; Rounsaville, B.J.; Kosten, T.R.; Jatlow, P.I.; Morgan, C. Desipramine Facilitation of Initial Cocaine Abstinence. *Arch. Gen. Psychiatry* **1989**, *46*, 117–121. https://doi.org/10.1001/archpsyc.1989.01810020019004.

27. Grabowski, J.; Rhoades, H.; Elk, R.; Schmitz, J.; Davis, C.; Creson, D.; Kirby, K. Fluoxetine Is Ineffective for Treatment of Cocaine Dependence or Concurrent Opiate and Cocaine Dependence: Two placebo-controlled double-blind trials. J. Clin. Psychopharmacol. 1995, 15, 163–174. https://doi.org/10.1097/00004714-199506000-00004.
28. Harris, D.S.; Batki, S.L.; Berger, S.P. Fluoxetine Attenuates Adrenocortical but Not Subjective Responses to Cocaine Cues. *Am. J. Drug Alcohol Abus.* **2004**, *30*, 765–782. https://doi.org/10.1081/ada-200037542.
29. Kolar, A.F.; Brown, B.S.; Weddington, W.W.; Haertzen, C.C.; Michaelson, B.S.; Jaffe, J.H. Treatment of Cocaine Dependence in Methadone Maintenance Clients: A Pilot Study Comparing the Efficacy of Desipramine and Amantadine. *Int. J. Addict.* **1992**, *27*, 849–868. https://doi.org/10.3109/10826089209068770.
30. Kosten, T.R.; Morgan, C.M.; Falcione, J.; Schottenfeld, R.S. Pharmacotherapy for Cocaine-Abusing Methadone-Maintained Patients Using Amantadine or Desipramine. *Arch. Gen. Psychiatry* **1992**, *49*, 894–898. https://doi.org/10.1001/archpsyc.1992.01820110058009.
31. McDowell, D.; Nunes, E.V.; Seracini, A.M.; Rothenberg, J.; Vosburg, S.K.; Ma, G.J.; Petkova, E. Desipramine treatment of cocaine-dependent patients with depression: A placebo-controlled trial. *Drug Alcohol Depend.* **2005**, *80*, 209–221. https://doi.org/10.1016/j.drugalcdep.2005.03.026.
33. Oliveto, A.; Kosten, T.R.; Schottenfeld, R.; Falcioni, J.; Ziedonis, D. Desipramine, amantadine, or fluoxetine in buprenorphine-maintained cocaine users. *J. Subst. Abus. Treat.* **1995**, *12*, 423–428. https://doi.org/10.1016/0740-547202015-2.
34. Passos, S.R.L.; Camacho, L.A.B.; Lopes, C.D.S.; Dos Santos, M.A.B. Nefazodone in out-patient treatment of inhaled cocaine dependence: A randomized double-blind placebo-controlled trial. *Addiction* **2005**, *100*, 489–494. https://doi.org/10.1111/j.1360-0443.2005.01041.x.
35. Shoptaw, S.; Heinzerling, K.G.; Rotheram-Fuller, E.; Kao, U.H.; Wang, P.-C.; Bholat, M.A.; Ling, W. Bupropion Hydrochloride versus Placebo, in Combination with Cognitive Behavioral Therapy, for the Treatment of Cocaine Abuse/Dependence. *J. Addict. Dis.* **2008**, *27*, 13–23. https://doi.org/10.1300/j069v27n01_02.
37. Alim, T.N.; Rosse, R.B.; Vocci, F.J.; Lindquist, T.; Deutsch, S.I. Diethylpropion Pharmacotherapeutic Adjuvant Therapy for Inpatient Treatment of Cocaine Dependence: A test of the cocaine-agonist hypothesis. *Clin. Neuropharmacol.* **1995**, *18*, 183–195. https://doi.org/10.1097/00002826-199504000-00009.
38. Anderson, A.L.; Reid, M.S.; Li, S.-H.; Holmes, T.; Shemanski, L.; Slee, A.; Smith, E.V.; Kahn, R.; Chiang, N.; Vocci, F.; et al. Modafinil for the treatment of cocaine dependence. *Drug Alcohol Depend.* **2009**, *104*, 133–139. https://doi.org/10.1016/j.drugalcdep.2009.04.015.
39. Buydens-Branchey, L.; Branchey, M.; Fergeson, P.; Hudson, J.; McKernin, C. Craving for cocaine in addicted users. Role of serotonergic mechanisms. *Am. J. Addict.* **1997**, *6*, 65–73.
40. Cantilena, L.; Kahn, R.; Duncan, C.C.; Li, S.-H.; Anderson, A.; Elkashef, A. Safety of Atomoxetine in Combination With Intravenous Cocaine in Cocaine-Experienced Participants. *J. Addict. Med.* **2012**, *6*, 265–273. https://doi.org/10.1097/adm.0b013e31826b767f.
41. Cornish, J.W.; Maany, I.; Fudala, P.J.; Ehrman, R.N.; Robbins, S.J.; O'Brien, C.P. A randomized, double-blind, placebo-controlled study of ritanserin pharmacotherapy for cocaine dependence. *Drug Alcohol Depend.* **2000**, *61*, 183–189. https://doi.org/10.1016/s0376-871600140-x.
42. Dackis, C.A.; Lynch, K.G.; Yu, E.; Samaha, F.F.; Kampman, K.M.; Cornish, J.W.; Rowan, A.; Poole, S.; White, L.; O'Brien, C.P. Modafinil and cocaine: A double-blind, placebo-controlled drug interaction study. *Drug Alcohol Depend.* **2002**, *70*, 29–37. https://doi.org/10.1016/s0376-871600335-6.
43. Dackis, C.A.; Kampman, K.M.; Lynch, K.G.; Pettinati, H.M.; O'Brien, C.P. A Double-Blind, Placebo-Controlled Trial of Modafinil for Cocaine Dependence. *Neuropsychopharmacology* **2004**, *30*, 205–211. https://doi.org/10.1038/sj.npp.1300600.
44. Dackis, C.A.; Kampman, K.M.; Lynch, K.G.; Plebani, J.G.; Pettinati, H.M.; Sparkman, T.; O'Brien, C.P. A double-blind, placebo-controlled trial of modafinil for cocaine dependence. *J. Subst. Abus. Treat.* **2012**, *43*, 303–312. https://doi.org/10.1016/j.jsat.2011.12.014.
45. Ehrman, R.N.; Robbins, S.J.; Cornish, J.W.; Childress, A.R.; O'Brien, C.P. Failure of ritanserin to block cocaine cue reactivity in humans. *Drug Alcohol Depend.* **1996**, *42*, 167–174. https://doi.org/10.1016/s0376-871601278-1.
46. Goudriaan, A.E.; Veltman, D.J.; Brink, W.V.D.; Dom, G.; Schmaal, L. Neurophysiological effects of modafinil on cue-exposure in cocaine dependence: A randomized placebo-controlled cross-over study using pharmacological fMRI. Addict. Behav. 2013, 38, 1509–1517. https://doi.org/10.1016/j.addbeh.2012.04.006.
47. Grabowski, J.; Roache, J.D.; Schmitz, J.; Rhoades, H.; Creson, D.; Korszun, A. Replacement Medication for Cocaine Dependence: Methylphenidate. J. Clin. Psychopharmacol. 1997, 17, 485–488. https://doi.org/10.1097/00004714-199712000-00008.
48. Greenwald, M.K.; Lundahl, L.H.; Steinmiller, C.L. Sustained Release d-Amphetamine Reduces Cocaine but not 'Speedball'-Seeking in Buprenorphine-Maintained Volunteers: A Test of Dual-Agonist Pharmacotherapy for Cocaine/Heroin Polydrug Abusers. *Neuropsychopharmacology* **2010**, *35*, 2624–2637. https://doi.org/10.1038/npp.2010.175.

50. Johnson, B.A.; Chen, Y.R.; Swann, A.C.; Schmitz, J.; Lesser, J.; Ruiz, P.; Johnson, P.; Clyde, C. Ritanserin in the treatment of cocaine dependence. *Biol. Psychiatry* **1997**, *42*, 932–940. https://doi.org/10.1016/s0006-322300490-8.
51. Kampman, K.M.; Lynch, K.G.; Pettinati, H.M.; Spratt, K.; Wierzbicki, M.R.; Dackis, C.; O'Brien, C.P. A double blind, placebo controlled trial of modafinil for the treatment of cocaine dependence without co-morbid alcohol dependence. *Drug Alcohol Depend.* **2015**, *155*, 105–110. https://doi.org/10.1016/j.drugalcdep.2015.08.005.
52. Karila, L.; Leroy, C.; Dubol, M.; Trichard, C.; Mabondo, A.; Marill, C.; Dubois, A.; Bordas, N.; Martinot, J.-L.; Reynaud, M.; et al. Dopamine Transporter Correlates and Occupancy by Modafinil in Cocaine-Dependent Patients: A Controlled Study With High-Resolution PET and [11C]-PE2I. *Neuropsychopharmacology* **2016**, *41*, 2294–2302. https://doi.org/10.1038/npp.2016.28.
53. Levin, F.R.; Evans, S.M.; Brooks, D.J.; Garawi, F. Treatment of cocaine dependent treatment seekers with adult ADHD: Double-blind comparison of methylphenidate and placebo. *Drug Alcohol Depend.* **2007**, *87*, 20–29. https://doi.org/10.1016/j.drugalcdep.2006.07.004.
54. Mooney, M.E.; Herin, D.V.; Schmitz, J.; Moukaddam, N.; Green, C.E.; Grabowski, J. Effects of oral methamphetamine on cocaine use: A randomized, double-blind, placebo-controlled trial. *Drug Alcohol Depend.* **2009**, *101*, 34–41. https://doi.org/10.1016/j.drugalcdep.2008.10.016.
55. Mooney, M.E.; Herin, D.V.; Specker, S.; Babb, D.; Levin, F.R.; Grabowski, J. Pilot study of the effects of lisdexamfetamine on cocaine use: A randomized, double-blind, placebo-controlled trial. *Drug Alcohol Depend.* **2015**, *153*, 94–103. https://doi.org/10.1016/j.drugalcdep.2015.05.042.
56. Nuijten, M.; Blanken, P.; van de Wetering, B.; Nuijen, B.; Brink, W.V.D.; Hendriks, V.M. Sustained-release dexamfetamine in the treatment of chronic cocaine-dependent patients on heroin-assisted treatment: A randomised, double-blind, placebo-controlled trial. *Lancet* **2016**, *387*, 2226–2234. https://doi.org/10.1016/s0140-673600205-1.
57. Perry, E.B.; Gil, R.; Miles, D.; Brenner, L.; MacDougall, L.; Johnson, R.; Degen, K.; Gueorguieva, R.; Petrakis, I.L.; Krystal, J.H.; et al. Mazindol Augmentation of Antipsychotic Treatment for Schizophrenic Patients with Comorbid Cocaine Abuse or Dependence. *J. Dual Diagn.* **2005**, *1*, 37–47. https://doi.org/10.1300/j374v01n01_04.
58. Pirtle, J.L.; Hickman, M.D.; Boinpelly, V.C.; Surineni, K.; Thakur, H.K.; Grasing, K.W. The serotonin-2C agonist Lorcaserin delays intravenous choice and modifies the subjective and cardiovascular effects of cocaine: A randomized, controlled human laboratory study. *Pharmacol. Biochem. Behav.* **2019**, *180*, 52–59. https://doi.org/10.1016/j.pbb.2019.02.010.
59. Roache, J.D.; Grabowski, J.; Schmitz, J.M.; Creson, D.L.; Rhoades, H.M. Laboratory Measures of Methylphenidate Effects in Cocaine-Dependent Patients Receiving Treatment. *J. Clin. Psychopharmacol.* **2000**, *20*, 61–68. https://doi.org/10.1097/00004714-200002000-00011.
61. Schubiner, H.; Downey, K.K.; Arfken, C.L.; Johanson, C.-E.; Schuster, C.R.; Lockhart, N.; Edwards, A.; Donlin, J.; Pihlgren, E. Double-blind placebo-controlled trial of methylphenidate in the treatment of adult ADHD patients with comorbid cocaine dependence.. *Exp. Clin. Psychopharmacol.* **2002**, *10*, 286–294. https://doi.org/10.1037//1064-1297.10.3.286.
62. Shearer, J.; Wodak, A.; Van Beek, I.; Mattick, R.P.; Lewis, J. Pilot randomized double blind placebo-controlled study of dexamphetamine for cocaine dependence. *Addiction* **2003**, *98*, 1137–1141. https://doi.org/10.1046/j.1360-0443.2003.00447.x.
63. Stine, S.M.; Krystal, J.H.; Kosten, T.R.; Charney, D.S. Mazindol treatment for cocaine dependence. *Drug Alcohol Depend.* **1995**, *39*, 245–252. https://doi.org/10.1016/0376-871601174-4.
64. Walsh, S.L.; Middleton, L.S.; Wong, C.J.; Nuzzo, P.A.; Campbell, C.L.; Rush, C.R.; Lofwall, M.R. Atomoxetine does not alter cocaine use in cocaine dependent individuals: A double blind randomized trial. *Drug Alcohol Depend.* **2012**, *130*, 150–157. https://doi.org/10.1016/j.drugalcdep.2012.10.024.
65. Akerele, E.; Levin, F.R. Comparison of Olanzapine to Risperidone in Substance-Abusing Individuals with Schizophrenia. *Am. J. Addict.* **2007**, *16*, 260–268. https://doi.org/10.1080/10550490701389658.
66. Beresford, T.; Buchanan, J.; Thumm, E.B.; Emrick, C.; Weitzenkamp, D.; Ronan, P.J. Late Reduction of Cocaine Cravings in a Randomized, Double-Blind Trial of Aripiprazole vs Perphenazine in Schizophrenia and Comorbid Cocaine Dependence. *J. Clin. Psychopharmacol.* **2017**, *37*, 657–663. https://doi.org/10.1097/jcp.0000000000000789.
67. Hamilton, J.D.; Nguyen, Q.X.; Gerber, R.M.; Rubio, N.B. Olanzapine in Cocaine Dependence: A Double-Blind, Placebo-Controlled Trial. *Am. J. Addict.* **2009**, *18*, 48–52. https://doi.org/10.1080/10550490802544318.
68. Kampman, K.M.; Pettinati, H.; Lynch, K.G.; Sparkman, T.; O'Brien, C.P. A pilot trial of olanzapine for the treatment of cocaine dependence. *Drug Alcohol Depend.* **2003**, *70*, 265–273. https://doi.org/10.1016/s0376-871600009-7.
69. Loebl, T.; Angarita, G.A.; Pachas, G.N.; Huang, K.-L.; Lee, S.H.; Nino, J.; Logvineko, T.; Culhane, M.A.; Evins, A.E. A Randomized, Double-Blind, Placebo-Controlled Trial of Long-Acting Risperidone in Cocaine-Dependent Men. *J. Clin. Psychiatry* **2008**, *69*, 480–486. https://doi.org/10.4088/jcp.v69n0321.

70. Lofwall, M.R.; Nuzzo, P.A.; Campbell, C.; Walsh, S.L. Aripiprazole effects on self-administration and pharmacodynamics of intravenous cocaine and cigarette smoking in humans.. *Exp. Clin. Psychopharmacol.* **2014**, *22*, 238–247. https://doi.org/10.1037/a0035165.
71. Moran, L.M.; Phillips, K.A.; Kowalczyk, W.; Ghitza, U.E.; Agage, D.A.; Epstein, D.H.; Preston, K.L. Aripiprazole for cocaine abstinence: A randomized–controlled trial with ecological momentary assessment. *Behav. Pharmacol.* **2017**, *28*, 63–73. https://doi.org/10.1097/fbp.0000000000000268.
72. Reid, M.S.; Casadonte, P.; Baker, S.; Sanfilipo, M.; Braunstein, D.; Hitzemann, R.; Montgomery, A.; Majewska, D.; Robinson, J.; Rotrosen, J. A placebo-controlled screening trial of olanzapine, valproate, and coenzyme Q10/L-carnitine for the treatment of cocaine dependence. *Addiction* **2005**, *100*, 43–57. https://doi.org/10.1111/j.1360-0443.2005.00990.x.
73. Sayers, S.L.; Campbell, E.C.; Kondrich, J.; Mann, S.C.; Cornish, J.; O'Brien, C.; Caroff, S.N. Cocaine Abuse in Schizophrenic Patients Treated With Olanzapine Versus Haloperidol. *J. Nerv. Ment. Dis.* **2005**, *193*, 379–386. https://doi.org/10.1097/01.nmd.0000165089.14736.bf.
74. Smelson, D.A.; Williams, J.; Ziedonis, D.; Sussner, B.D.; Losonczy, M.F.; Engelhart, C.; Kaune, M. A double-blind placebo-controlled pilot study of risperidone for decreasing cue-elicited craving in recently withdrawn cocaine dependent patients. *J. Subst. Abus. Treat.* **2004**, *27*, 45–49. https://doi.org/10.1016/j.jsat.2004.03.009.
75. Smelson, D.A.; Ziedonis, D.; Williams, J.; Losonczy, M.F.; Williams, J.; Steinberg, M.L.; Kaune, M. The Efficacy of Olanzapine for Decreasing Cue-Elicited Craving in Individuals With Schizophrenia and Cocaine Dependence: A preliminary report. *J. Clin. Psychopharmacol.* **2006**, *26*, 9–12. https://doi.org/10.1097/01.jcp.0000194624.07611.5e.
76. Tapp, A.; Wood, A.E.; Kennedy, A.; Sylvers, P.; Kilzieh, N.; Saxon, A.J. Quetiapine for the treatment of cocaine use disorder. *Drug Alcohol Depend.* **2015**, *149*, 18–24. https://doi.org/10.1016/j.drugalcdep.2014.12.037.
77. Bisaga, A.; Aharonovich, E.; Garawi, F.; Levin, F.R.; Rubin, E.; Raby, W.N.; Nunes, E. A randomized placebo-controlled trial of gabapentin for cocaine dependence. *Drug Alcohol Depend.* **2006**, *81*, 267–274. https://doi.org/10.1016/j.drugalcdep.2005.07.009.
78. Brown, E.S.; Sunderajan, P.; Hu, L.T.; Sowell, S.M.; Carmody, T.J. A Randomized, Double-Blind, Placebo-Controlled, Trial of Lamotrigine Therapy in Bipolar Disorder, Depressed or Mixed Phase and Cocaine Dependence. *Neuropsychopharmacology* **2012**, *37*, 2347–2354. https://doi.org/10.1038/npp.2012.90.
79. Crosby, R.; Pearson, V.L.; Eller, C.; Winegarden, T.; Graves, N.L. Phenytoin in the treatment of cocaine abuse: A double-blind study*. *Clin. Pharmacol. Ther.* **1996**, *59*, 458–468. https://doi.org/10.1016/s0009-923690116-2.
80. Halikas, J.A.; Crosby, R.D.; Pearson, V.L.; Graves, N.M. A randomized double-blind study of carbamazepine in the treatment of cocaine abuse*. *Clin. Pharmacol. Ther.* **1997**, *62*, 89–105. https://doi.org/10.1016/s0009-923690155-7.
81. Johnson, B.A.; Roache, J.D.; Ait-Daoud, N.; Gunderson, E.W.; Haughey, H.M.; Wang, X.-Q.; Liu, L. Topiramate's effects on cocaine-induced subjective mood, craving and preference for money over drug taking.. *Addict. Biol.* **2012**, *18*, 405–416,. https://doi.org/10.1111/j.1369-1600.2012.00499.x.
83. Kampman, K.M.; Pettinati, H.M.; Lynch, K.G.; Spratt, K.; Wierzbicki, M.R.; O'Brien, C.P. A double-blind, placebo-controlled trial of topiramate for the treatment of comorbid cocaine and alcohol dependence. *Drug Alcohol Depend.* **2013**, *133*, 94–99. https://doi.org/10.1016/j.drugalcdep.2013.05.026.
84. Reid, M.S.; Thakkar, V. Valproate treatment and cocaine cue reactivity in cocaine dependent individuals. *Drug Alcohol Depend.* **2009**, *102*, 144–150. https://doi.org/10.1016/j.drugalcdep.2009.02.010.
85. Reid, M.S.; Mickalian, J.D.; Delucchi, K.L.; Berger, S.P. A nicotine antagonist, mecamylamine, reduces cue-induced cocaine craving in cocaine-dependent subjects.. *Neuropsychopharmacology* **1999**, *20*, 297–307. https://doi.org/10.1016/S0893-133X(98)00076-1.
86. Umbricht, A.; DeFulio, A.; Winstanley, E.L.; Tompkins, D.A.; Peirce, J.; Mintzer, M.Z.; Strain, E.C.; Bigelow, G.E. Topiramate for cocaine dependence during methadone maintenance treatment: A randomized controlled trial. *Drug Alcohol Depend.* **2014**, *140*, 92–100. https://doi.org/10.1016/j.drugalcdep.2014.03.033.
87. Johnson, B.A.; Ait-Daoud, N.; Wang, X.-Q.; Penberthy, J.K.; Javors, M.A.; Seneviratne, C.; Liu, L. Topiramate for the Treatment of Cocaine Addiction: A randomized clinical trial. *JAMA Psychiatry* **2013**, *70*, 1338–1346. https://doi.org/10.1001/jamapsychiatry.2013.2295.
87. Winhusen, T.; Somoza, E.; Ciraulo, D.A.; Harrer, J.M.; Goldsmith, R.J.; Grabowski, J.; Coleman, F.S.; Mindrum, G.; Kahn, R.; Osman, S.; et al. A double-blind, placebo-controlled trial of tiagabine for the treatment of cocaine dependence. *Drug Alcohol Depend.* **2007**, *91*, 141–148. https://doi.org/10.1016/j.drugalcdep.2007.05.028.
88. Becker, J.; Price, J.L.; Leonard, D.; Suris, A.; Kandil, E.; Shaw, M.; Kroener, S.; Brown, E.S.; Adinoff, B. The Efficacy of Lidocaine in Disrupting Cocaine Cue-Induced Memory Reconsolidation. *Drug Alcohol Depend.* **2020**, *212*, 108062. https://doi.org/10.1016/j.drugalcdep.2020.108062.
89. Bisaga, A.; Aharonovich, E.; Cheng, W.Y.; Levin, F.R.; Mariani, J.J.; Raby, W.N.; Nunes, E. A placebo-controlled trial of memantine for cocaine dependence with high-value voucher incentives during a pre-

randomization lead-in period. *Drug Alcohol Depend.* **2010**, *111*, 97–104. https://doi.org/10.1016/j.drugalcdep.2010.04.006.

90. Brown, E.S.; Todd, J.P.; Hu, L.T.; Schmitz, J.M.; Carmody, T.J.; Nakamura, A.; Sunderajan, P.; Rush, A.J.; Adinoff, B.; Bret, M.E.; et al. A Randomized, Double-Blind, Placebo-Controlled Trial of Citicoline for Cocaine Dependence in Bipolar I Disorder. *Am. J. Psychiatry* **2015**, *172*, 1014–1021. https://doi.org/10.1176/appi.ajp.2015.14070857.
91. Chadwick, M.J.; Gregory, D.L.; Wendling, G. A Double-Blind Amino Acids, L-Tryptophan and L-Tyrosine, and Placebo Study with Cocaine-Dependent Subjects in an Inpatient Chemical Dependency Treatment Center. *Am. J. Drug Alcohol Abus.* **1990**, *16*, 275–286. https://doi.org/10.3109/00952999009001589.
92. Dackis, C.A.; Gold, M.S.; Sweeney, D.R.; Byron, J.P.; Climko, R. Single-dose bromocriptine reverses cocaine craving. *Psychiatry Res.* **1987**, *20*, 261–264. https://doi.org/10.1016/0165-178190086-2.
93. Dakwar, E.; Levin, F.; Foltin, R.W.; Nunes, E.V.; Hart, C.L. The Effects of Subanesthetic Ketamine Infusions on Motivation to Quit and Cue-Induced Craving in Cocaine-Dependent Research Volunteers. Biol. Psychiatry 2014, 76, 40–46. https://doi.org/10.1016/j.biopsych.2013.08.009.
94. Dakwar, E.; Anerella, C.; Hart, C.; Levin, F.; Mathew, S.; Nunes, E. Therapeutic infusions of ketamine: Do the psychoactive effects matter?. *Drug Alcohol Depend.* **2014**, *136*, 153–157. https://doi.org/10.1016/j.drugalcdep.2013.12.019.
95. Dakwar, E.; Hart, C.L.; Levin, F.R.; Nunes, E.V.; Foltin, R.W. Cocaine self-administration disrupted by the N-methyl-D-aspartate receptor antagonist ketamine: A randomized, crossover trial. Mol. Psychiatry 2016, 22, 76–81. https://doi.org/10.1038/mp.2016.39.
96. Dieckmann, L.H.J.; Ramos, A.C.; Silva, E.A.; Justo, L.P.; Sabioni, P.; Frade, I.F.; de Souza, A.L.; Galduróz, J.C.F. Effects of biperiden on the treatment of cocaine/crack addiction: A randomised, double-blind, placebo-controlled trial. *Eur. Neuropsychopharmacol.* **2014**, *24*, 1196–1202. https://doi.org/10.1016/j.euroneuro.2014.06.001.
97. Eiler, K.; Schaefer, M.R.; Salstrom, D.; Lowery, R. Double-Blind Comparison of Bromocriptine and Placebo in Cocaine Withdrawal. *Am. J. Drug Alcohol Abus.* **1995**, *21*, 65–79. https://doi.org/10.3109/00952999509095230.
98. Elkashef, A.; Fudala, P.J.; Gorgon, L.; Li, S.-H.; Kahn, R.; Chiang, N.; Vocci, F.; Collins, J.; Jones, K.; Boardman, K.; et al. Double-blind, placebo-controlled trial of selegiline transdermal system (STS) for the treatment of cocaine dependence. *Drug Alcohol Depend.* **2006**, *85*, 191–197. https://doi.org/10.1016/j.drugalcdep.2006.04.010.
99. Fox, H.C.; Seo, D.; Tuit, K.; Hansen, J.; Kimmerling, A.; Morgan, P.T.; Sinha, R. Guanfacine effects on stress, drug craving and prefrontal activation in cocaine dependent individuals: Preliminary findings. *J. Psychopharmacol.* **2012**, , 958–972. https://doi.org/10.1177/0269881111430746.

Renshaw, P.F.; Daniels, S.; Lundahl, L.H.; Rogers, V.; Lukas, S.E. Short-term treatment with citicoline (CDP-choline) attenuates some measures of craving in cocaine-dependent subjects: A preliminary report.. *Psychopharmacology* **1999**, *142*, 132–138. https://doi.org/10.1007/s002130050871.

100. Fox, H.C.; Sofuoglu, M.; Morgan, P.T.; Tuit, K.L.; Sinha, R. The effects of exogenous progesterone on drug craving and stress arousal in cocaine dependence: Impact of gender and cue type. *Psychoneuroendocrinology* **2013**, *38*, 1532–1544. https://doi.org/10.1016/j.psyneuen.2012.12.022.
101. Handelsman, L.; Limpitlaw, L.; Williams, D.; Schmeidler, J.; Paris, P.; Stimmel, B. Amantadine does not reduce cocaine use or craving in cocaine-dependent methadone maintenance patients. *Drug Alcohol Depend.* **1995**, *39*, 173–180. https://doi.org/10.1016/0376-871601154-9.
102. Handelsman, L.; Rosenblum, A.; Palij, M.; Magura, S.; Foote, J.; Lovejoy, M.; Stimmel, B. Bromocriptine for cocaine de-pendence. A controlled clinical trial. *Am. J. Addict.* **1997**, *6*, 54–64.
103. Jobes, M.L.; Ghitza, U.E.; Epstein, D.H.; Phillips, K.A.; Heishman, S.J.; Preston, K.L. Clonidine blocks stress-induced craving in cocaine users. *Psychopharmacology* **2011**, *218*, 83–88. https://doi.org/10.1007/s00213-011-2230-7.
104. Jobes, M.L.; Aharonovich, E.; Epstein, D.H.; Phillips, K.A.; Reamer, D.; Anderson, M.; Preston, K.L. Effects of Prereactivation Propranolol on Cocaine Craving Elicited by Imagery Script/Cue Sets in Opioid-dependent Polydrug Users: A Ran-domized Study. *J. Addict. Med.* **2015**, *9*, 491–498. https://doi.org/10.1097/adm.0000000000000169.
105. Johnson, B.A.; Roache, J.D.; Ait-Daoud, N.; Wells, L.T.; Mauldin, J.B. Effects of Isradipine on Cocaine-Induced Subjective Mood. *J. Clin. Psychopharmacol.* **2004**, *24*, 180–191. https://doi.org/10.1097/01.jcp.0000115662.45074.c3.
106. Johnson, M.W.; Bruner, N.R.; Johnson, P.S.; Silverman, K.; Berry, M.S. Randomized controlled trial of d-cycloserine in cocaine dependence: Effects on contingency management and cue-induced cocaine craving in a naturalistic setting.. *Exp. Clin. Psychopharmacol.* **2020**, *28*, 157–168. https://doi.org/10.1037/pha0000306.
107. Kablinger, A.S.; Lindner, M.A.; Casso, S.; Hefti, F.; DeMuth, G.; Fox, B.S.; McNair, L.; McCarthy, B.G.; Goeders, N.E. Effects of the combination of metyrapone and oxazepam on cocaine craving and cocaine

taking: A double-blind, randomized, placebo-controlled pilot study. *J. Psychopharmacol.* **2012**, *26*, 973–981. https://doi.org/10.1177/0269881111430745.
108. Kampman, K.M.; Volpicelli, J.R.; Mulvaney, F.; Alterman, A.I.; Cornish, J.; Gariti, P.; Cnaan, A.; Poole, S.; Muller, E.; Acosta, T.; et al. Effectiveness of propranolol for cocaine dependence treatment may depend on cocaine withdrawal symptom severity. *Drug Alcohol Depend.* **2001**, *63*, 69–78. https://doi.org/10.1016/s0376-871600193-9.
109. Kampman, K.M.; Dackis, C.; Lynch, K.G.; Pettinati, H.; Tirado, C.; Gariti, P.; Sparkman, T.; Atzram, M.; O'Brien, C.P. A double-blind, placebo-controlled trial of amantadine, propranolol, and their combination for the treatment of cocaine dependence in patients with severe cocaine withdrawal symptoms. *Drug Alcohol Depend.* **2006**, *85*, 129–137. https://doi.org/10.1016/j.drugalcdep.2006.04.002.
110. Winhusen, T.; Somoza, E.; Harrer, J.; Moore, E.; Ussery, T.; Kropp, F.; Singal, B.; Elkashef, A.; Mojsiak, J. Metyrapone and cocaine: A double-blind, placebo-controlled drug interaction study. *Pharmacol. Biochem. Behav.* **2005**, *80*, 631–638. https://doi.org/10.1016/j.pbb.2005.01.017.
111. Lee, M.R.; Glassman, M.; King-Casas, B.; Kelly, D.L.; Stein, E.A.; Schroeder, J.; Salmeron, B.J. Complexity of oxytocin′s effects in a chronic cocaine dependent population. *Eur. Neuropsychopharmacol.* **2014**, *24*, 1483–1491. https://doi.org/10.1016/j.euroneuro.2014.06.005.
112. Leyton, M.; Casey, K.F.; Delaney, J.S.; Kolivakis, T.; Benkelfat, C. Cocaine craving, euphoria, and self-administration: A preliminary study of the effect of catecholamine precursor depletion.. *Behav. Neurosci.* **2005**, *119*, 1619–1627. https://doi.org/10.1037/0735-7044.119.6.1619.
113. Licata, S.C.; Penetar, D.M.; Ravichandran, C.; Rodolico, J.; Palmer, C.; Berko, J.; Geaghan, T.; Looby, A.; Peters, E.; Ryan, E.; et al. Effects of Daily Treatment With Citicoline: A Double-Blind, Placebo-Controlled Study in Cocaine-Dependent Volunteers. *J. Addict. Med.* **2011**, *5*, 57–64. https://doi.org/10.1097/adm.0b013e3181d80c93.
114. Malcolm, R.; Kajdasz, D.K.; Herron, J.; Anton, R.F.; Brady, K.T. A double-blind, placebo-controlled outpatient trial of pergolide for cocaine dependence. *Drug Alcohol Depend.* **2000**, *60*, 161–168. https://doi.org/10.1016/s0376-871600151-9.
115. Malcolm, R.; LaRowe, S.; Cochran, K.; Moak, D.; Herron, J.; Brady, K.; Hedden, S.; Woolson, R.; Halushka, P. A controlled trial of amlodipine for cocaine dependence: A negative report. *J. Subst. Abus. Treat.* **2005**, *28*, 197–204. https://doi.org/10.1016/j.jsat.2004.12.006.
117. Milivojevic, V.; Fox, H.C.; Sofuoglu, M.; Covault, J.; Sinha, R. Effects of progesterone stimulated allopregnanolone on craving and stress response in cocaine dependent men and women. *Psychoneuroendocrinology* **2016**, *65*, 44–53. https://doi.org/10.1016/j.psyneuen.2015.12.008.
118. Modesto-Lowe, V.; Burleson, J.A.; Hersh, D.; Bauer, L.O.; Kranzler, H.R. Effects of naltrexone on cue-elicited craving for alcohol and cocaine. *Drug Alcohol Depend.* **1997**, *49*, 9–16. https://doi.org/10.1016/s0376-871600134-8.
120. Mooney, M.E.; Schmitz, J.; Moeller, F.G.; Grabowski, J. Safety, tolerability and efficacy of levodopa–carbidopa treatment for cocaine dependence: Two double-blind, randomized, clinical trials. *Drug Alcohol Depend.* **2007**, *88*, 214–223. https://doi.org/10.1016/j.drugalcdep.2006.10.011.
121. Maria, M.M.M.-S.; Baker, N.L.; Ramakrishnan, V.; Brady, K.T.; McRae-Clark, A. Impact of acute guanfacine administration on stress and cue reactivity in cocaine-dependent individuals. *Am. J. Drug Alcohol Abus.* **2014**, *41*, 146–152. https://doi.org/10.3109/00952990.2014.945590.
122. Nann-Vernotica, E.; Donny, E.; Bigelow, G.E.; Walsh, S.L. Repeated administration of the D 1/5 antagonist ecopipam fails to attenuate the subjective effects of cocaine. *Psychopharmacology* **2001**, *155*, 338–347. https://doi.org/10.1007/s002130100724.
123. Nasser, A.F.; Fudala, P.J.; Zheng, B.; Liu, Y.; Heidbreder, C. A Randomized, Double-Blind, Placebo-Controlled Trial of RBP-8000 in Cocaine Abusers: Pharmacokinetic Profile of RBP-8000 and Cocaine and Effects of RBP-8000 on Cocaine-Induced Physiological Effects. *J. Addict. Dis.* **2014**, *33*, 289–302. https://doi.org/10.1080/10550887.2014.969603.
124. Newton, T.F.; Haile, C.N.; Mahoney, J.J.; Shah, R.; Verrico, C.D.; De La Garza, R.; Kosten, T.R. Dopamine D3 receptor-preferring agonist enhances the subjective effects of cocaine in humans. *Psychiatry Res.* **2015**, *230*, 44–49. https://doi.org/10.1016/j.psychres.2015.07.073.
125. Petrakis, I.L.; Carroll, K.; Nich, C.; Gordon, L.T.; McCance-Katz, E.F.; Frankforter, T.; Rounsaville, B.J. Disulfiram treatment for cocaine dependence in methadone-maintained opioid addicts. *Addiction* **2000**, *95*, 219–228. https://doi.org/10.1046/j.1360-0443.2000.9522198.x.
126. Plebani, J.G.; Lynch, K.G.; Yu, Q.; Pettinati, H.M.; O'Brien, C.P.; Kampman, K.M. Results of an initial clinical trial of varenicline for the treatment of cocaine dependence. *Drug Alcohol Depend.* **2012**, *121*, 163–166. https://doi.org/10.1016/j.drugalcdep.2011.08.025.
127. Preston, K.; Sullivan, J.T.; Berger, P.; Bigelow, G.E. Effects of cocaine alone and in combination with mazindol in human cocaine abusers.. *J. Pharmacol. Exp. Ther.* **1993**, *267*, 296–307.

128. Price, K.L.; Baker, N.L.; McRae-Clark, A.L.; Saladin, M.E.; DeSantis, S.M.; Ana, E.J.S.; Brady, K.T. A randomized, placebo-controlled laboratory study of the effects of D-cycloserine on craving in cocaine-dependent individuals.. *Psychopharmacology* **2012**, *226*, 739–746,. https://doi.org/10.1007/s00213-011-2592-x.
129. Prisciandaro, J.J.; Myrick, H.; Henderson, S.; McRae-Clark, A.L.; Ana, E.J.S.; Saladin, M.E.; Brady, K.T. Impact of DCS-facilitated cue exposure therapy on brain activation to cocaine cues in cocaine dependence. *Drug Alcohol Depend.* **2013**, *132*, 195–201. https://doi.org/10.1016/j.drugalcdep.2013.02.009.
130. Reid, M.S.; Angrist, B.; Baker, S.; Woo, C.; Schwartz, M.; Montgomery, A.; Majewska, D.; Robinson, J.; Rotrosen, J. A placebo-controlled screening trial of celecoxib for the treatment of cocaine dependence. *Addiction* **2005**, *100*, 32–42. https://doi.org/10.1111/j.1360-0443.2005.00989.x.
131. Reid, M.S.; Angrist, B.; Ba, S.O.; Ba, J.S.; Schwartz, M.; Rotrosen, J.; Baker, S.A.; Robinson, J.; Leiderman, D.; Montgomery, A.; et al. A Placebo Controlled, Double-Blind Study of Mecamylamine Treatment for Cocaine Dependence in Patients Enrolled in an Opiate Replacement Program. *Subst. Abus.* **2006**, *26*, 5–14. https://doi.org/10.1300/j465v26n02_02.
133. Rosse, R.B.; Alim, T.N.; Fay-Mccarthy, M.; Collins, J.P.; Vocci, F.J.; Lindquist, T.; Jentgen, C.; Hess, A.L.; Deutsch, S.I. Nimodipine Pharmacotherapeutic Adjuvant Therapy for Inpatient Treatment of Cocaine Dependence. Clin. Neuropharmacol. 1994, 17, 348–358. https://doi.org/10.1097/00002826-199408000-00007.
134. Saladin, M.E.; Gray, K.M.; McRae-Clark, A.L.; LaRowe, S.D.; Yeatts, S.D.; Baker, N.L.; Hartwell, K.J.; Brady, K.T. A double blind, placebo-controlled study of the effects of post-retrieval propranolol on reconsolidation of memory for craving and cue reactivity in cocaine dependent humans. Psychopharmacology 2013, 226, 721–737. https://doi.org/10.1007/s00213-013-3039-3.
135.Ana, E.J.S.; Prisciandaro, J.J.; Saladin, M.E.; McRae-Clark, A.L.; Shaftman, S.R.; Nietert, P.J.; Brady, K.T. D-cycloserine combined with cue exposure therapy fails to attenuate subjective and physiological craving in cocaine dependence. *Am. J. Addict.* **2015**, *24*, 217–224. https://doi.org/10.1111/ajad.12191.
136. Schmitz, J.M.; Green, C.E.; Hasan, K.M.; Vincent, J.; Suchting, R.; Weaver, M.F.; Moeller, F.G.; Narayana, P.A.; Cunningham, K.A.; Dineley, K.T.; et al. PPAR-gamma agonist pioglitazone modifies craving intensity and brain white matter integrity in patients with primary cocaine use disorder: A double-blind randomized controlled pilot trial. *Addiction* **2017**, *112*, 1861–1868. https://doi.org/10.1111/add.13868.
137. Schmitz, J.M.; Stotts, A.L.; Rhoades, H.M.; Grabowski, J. Naltrexone and relapse prevention treatment for cocaine-dependent patients. *Addict. Behav.* **2001**, *26*, 167–180. https://doi.org/10.1016/s0306-460300098-8.
138. Schmitz, J.M.; Mooney, M.E.; Moeller, F.G.; Stotts, A.L.; Green, C.; Grabowski, J. Levodopa pharmacotherapy for cocaine dependence: Choosing the optimal behavioral therapy platform. *Drug Alcohol Depend.* **2008**, *94*, 142–150. https://doi.org/10.1016/j.drugalcdep.2007.11.004.
139. Schulte, M.H.; Wiers, R.; Boendermaker, W.J.; Goudriaan, A.E.; Brink, W.V.D.; van Deursen, D.S.; Friese, M.; Brede, E.; Waters, A.J. Reprint of The effect of N-acetylcysteine and working memory training on cocaine use, craving and inhibition in regular cocaine users: Correspondence of lab assessments and Ecological Momentary Assessment. *Addict. Behav.* **2018**, *83*, 79–86. https://doi.org/10.1016/j.addbeh.2018.03.023.
140. Shoptaw, S.; Kintaudi, P.; Charuvastra, C.; Ling, W. A screening trial of amantadine as a medication for cocaine dependence. *Drug Alcohol Depend.* **2001**, *66*, 217–224. https://doi.org/10.1016/s0376-871600205-8.
141. Shoptaw, S.; Yang, X.; Rotheram-Fuller, E.J.; Hsieh, Y.-C.M.; Kintaudi, P.C.; Charuvastra, V.C.; Ling, W. Randomized Placebo-Controlled Trial of Baclofen for Cocaine Dependence: Preliminary effects for individuals with chronic patterns of cocaine use. *J. Clin. Psychiatry* **2003**, *64*, 1440–1448. https://doi.org/10.4088/jcp.v64n1207.
142. Winhusen, T.; Somoza, E.; Sarid-Segal, O.; Goldsmith, R.J.; Harrer, J.M.; Coleman, F.S.; Kahn, R.; Osman, S.; Mezinskis, J.; Li, S.-H.; et al. A double-blind, placebo-controlled trial of reserpine for the treatment of cocaine dependence. *Drug Alcohol Depend.* **2007**, *91*, 205–212. https://doi.org/10.1016/j.drugalcdep.2007.05.021.
144. Somoza, E.C.; Winship, D.; Gorodetzky, C.W.; Lewis, D.; Ciraulo, D.A.; Galloway, G.P.; Segal, S.D.; Sheehan, M.; Roache, J.D.; Bickel, W.K.; et al. A Multisite, Double-blind, Placebo-Controlled Clinical Trial to Evaluate the Safety and Efficacy of Vigabatrin for Treating Cocaine Dependence. *JAMA Psychiatry* **2013**, *70*, 630–637. https://doi.org/10.1001/jamapsychiatry.2013.872.
145. Reid, M.S.; Mickalian, J.D.; Delucchi, K.L.; Hall, S.M.; Berger, S. An acute dose of nicotine enhances cue-induced cocaine craving. *Drug Alcohol Depend.* **1998**, *49*, 95–104. https://doi.org/10.1016/s0376-871600144-0.

146. Yonkers, K.A.; Forray, A.; Nich, C.; Carroll, K.M.; Hine, C.; Merry, B.C.; Shaw, H.; Shaw, J.; Sofuoglu, M. Progesterone for the reduction of cocaine use in post-partum women with a cocaine use disorder: A randomised, double-blind, placebo-controlled, pilot study. *Lancet Psychiatry* **2014**, *1*, 360–367. https://doi.org/10.1016/s2215-036670333-5.
147. Kampman, K.M.; Dackis, C.; Pettinati, H.M.; Lynch, K.G.; Sparkman, T.; O'Brien, C.P. A double-blind, placebo-controlled pilot trial of acamprosate for the treatment of cocaine dependence. *Addict. Behav.* **2011**, *36*, 217–221. https://doi.org/10.1016/j.addbeh.2010.11.003.
148. LaRowe, S.D.; Kalivas, P.W.; Nicholas, J.S.; Randall, P.K.; Mardikian, P.N.; Malcolm, R.J. A double-blind placebo-controlled trial of N-acetylcysteine in the treatment of cocaine dependence. *Am. J. Addict.* **2013**, *22*, 443–452. https://doi.org/10.1111/j.1521-0391.2013.12034.x.
